# From Past to Present: Biotechnology in Mexico Using Algae and Fungi

**DOI:** 10.3390/plants10112530

**Published:** 2021-11-20

**Authors:** Alvaro De Obeso Fernandez Del Valle, Christian Quintus Scheckhuber

**Affiliations:** Departamento de Bioingeniería, Escuela de Ingeniería y Ciencias, Tecnologico de Monterrey, Ave. Eugenio Garza Sada 2501, Monterrey 64849, Mexico; adeobeso@tec.mx

**Keywords:** algae, bioactive compounds, fungi, Mexico, microorganisms, pre-Hispanic

## Abstract

Algae and fungi share a rich history in the fields of basic and applied natural science. In biotechnology, in particular, algae and fungi are of paramount importance, due to the production and development of valuable compounds, such as pharmaceuticals, enzymes, and biofuels. They are also used in waste fermentation, biocontrol of pathogens, and food processing and improvement, among other fields. Although a substantial number of different microorganisms are utilized for these purposes, there lies tremendous potential in uncharacterized microbial species. For this reason, biodiversity hotspots offer a wealth of potential in the discovery of new products and processing strategies based on these microorganisms. This review presents an overview of the use of algae and fungi in pre-Hispanic times/modern-day Mexico for the benefits of mankind. One of our objectives is to raise awareness about the potential of developing research projects for identification and biotechnological utilization of algae and fungi in a megadiverse country, such as Mexico.

## 1. Introduction

Mexico is considered one of seventeen megadiverse countries in the world [1]. This diversity extends to algae and fungi, which have been used since pre-Hispanic times. However, the study of biodiversity is often neglected when it comes to the microbiological world. Deepening our knowledge about microorganisms is imperative toward utilizing them more efficiently for specific purposes. Unfortunately, although conservation efforts have been performed in Mexico since pre-Hispanic times, microorganisms are sometimes forgotten, as researchers instead focus on macroscopic diversity. These conservation efforts, performed in Mexico, have been documented and reviewed [2]. This review provides a short overview of the biotechnological utilization of algae and fungi, which are found in Mexico. First, we show that algae and fungi were important aspects of daily life in pre-Hispanic times. Then, we explore the field of biotechnology in modern-day Mexico, by outlining a few examples in which algae and fungi have been used in thematic fields, such as enzyme production, biocontrol strategies, food improvement, waste management, production of pharmaceutical compounds, and ludic uses. We finalize our review by providing a perspective on the potential innovations that a megadiverse country, such as Mexico, has to offer.

## 2. The Use of Algae and Fungi in Pre-Hispanic Mexico

The use of microorganisms in Mexico began before the Spanish conquest. Grains and fruit were fermented for thousands of years around the world, and Mexico was no exception [3]. Many beverages are still prepared nowadays using an assortment of different microorganisms. These beverages use several yeasts, including *Saccharomyces* and non-*Saccharomyces* species [4,5]. Although not conclusive, it has been suggested (and strong evidence points towards it) that pre-Hispanic cultures not only fermented beverages, but also distilled them [6,7]. 

Fermented Mexican beverages include “tejuino”, “pulque”, “pozol”, “tepache”, and bitter “atole”. These beverages have shown microbiological properties, such as probiotic capabilities, antibacterial, and fungicidal activities [8,9]. Several properties of fermented beverages in Mexico have been previously discussed [8,10]. One of the main examples is pulque, which is a fermented beverage from some agave species consumed in pre-Hispanic (and present) times in Mexico. Pulque includes probiotic organisms and was even adapted to be used as an enema to replenish the intestinal microbiota [11,12]. Another example, “pozol” is a non-alcoholic fermented beverage from native and mestizo cultures in Mexico. This beverage is produced from a form of processed maize called “nixtamal”. During fermentation, “nixtamal” changes the microbiota of the dough [13]. Empirically, this is evidence of pre-Hispanic cultures using microorganisms for their nutrition, health, and religious and ludic activities, among other purposes.

### 2.1. Algae

One of the most conspicuous groups of microorganisms used in pre-Hispanic Mexico was algae. Most information about algae use in native communities in Mexico comes from Mayan and Nahuatl communities (including the Aztecs) [14,15]. These cultures used micro-algae, mainly as dietary supplements. For example, the Aztecs of Texcoco used “tecuitlatl” (*Arthrospira platensis*), known today as spirulina, as a complement to maize [16]. Aztecs have consumed the blue–green algae since at least the 13th century [17].

Other algae commonly consumed in pre-Hispanic times include “cuculito”. “Cuculito” consists primarily of two algae: *Phormidium tenue* and *Chroococcus turgidus* [17]. “Cuculito” was used as a food supplement, which provided calcium and iron [18].

Aztecs also used “tizatl”, which consisted of diatoms from the Bacillariophyceae family. “Tizatl” was normally transformed as a white powder and commonly used for medicinal purposes [18]. Additionally, “tizatl” was frequently used as a white ornamental coloring for sacrificial war captives [19].

### 2.2. Fungi

The biological and cultural richness of fungi in Mexico is vast, but risks disappearing due to erosion of human cultures [20]. Several indigenous groups still use fungi in rituals, food, or medicine. The Mazatec, Nahuatl, Purepecha, Raramuris, and Zapotec are ancient Mesoamericans who were knowledgeable about the application of hallucinogenic fungi [21]. The most important hallucinogenic fungi belong to the genera *Psilocybe*, *Panaeolus*, and *Stropharia* [22]. Moreover, 3-[2-(dimethylamino)ethyl]-1H-indol-4-yl dihydrogen phosphate (psilocybin) is pharmacologically responsible for the hallucinogenic properties of *Psilocybe* spp., in which, once ingested, is converted to 4-hydroxy-N,N-dimethyltryptamine (psilocin), a potent hallucinogenic substance [23].

Consumption of hallucinogens in ceremonies and religious rituals spread from the valley of Mexico to all of Central America approximately 3500 years ago. The Mayas consumed a fungus known as “k’aizalaj okox” (*Psilocybe cubensis*), which was also known to the Aztecs, who named it “teonanacatl” [22]. This type of fungus was also consumed by the Huasteca, Totonac, Mazatec, and Mixtec. In Teotenango, one of their customs was to grind mushrooms with water on specialized stone plates of temples that were being constructed. Similar evidence was found in temples in other parts of Mexico and Guatemala, Honduras, and El Salvador [22]. The Mixtec God “Yya Sahuaco”, also known as the “Lord of Seven Flowers” is often represented with a pair of mushrooms in his hands. The mural of Tepantitla of Teotihuacán, dated to be created around AD 450, shows, below raindrops created by the Aztec rain god Tlaloc, the appearance of priests carrying (hallucinogenic) fungi. Furthermore, these fungi were employed at the coronation ceremonies of various Aztec emperors, including Tizoc, Ahuízotl, and Moctezuma II, who was ruler when Hernán Cortés arrived in 1519 on the east coast of the Aztec empire [22].

Fortunately, the traditional knowledge of indigenous people from different regions has been preserved, to a certain extent [24]. One of the intensely studied ethnicities are the Mixtec. The Mixtec use at least 26 fungal species for hallucinogenic, ludic, and medicinal applications [20]. In the Central Valleys of Oaxaca, there are twenty species of edible fungi being commercialized. These mushrooms are exported, have medicinal and nutritional value, and help as inoculants for forestry crops, such as prickly pear, *Opuntia* spp. [25,26]. Collectively, the use of mushrooms has been reported in 15 of the 68 indigenous groups and, in addition, in mestizo communities from rural areas [27,28].

Recently, the cultural importance of mushrooms among an indigenous group from the northern region of Jalisco, the Wixaritari, has been studied in detail [28]. In general, several mushroom species are highly valued as food, with low acceptance being very uncommon. Among the favored species are *Amanita basii* and *A. laurae*, *Volvariella bombycina*, *Pleurotus djamor,* and *P. opuntiae*. Galls from the smut *Ustilago maydis* are also appreciated as a source of food [28]. A few species serve ludic uses, e.g., fruiting bodies from *Calvatia cyathiformis* and *Pisolithus* spp. being used as projectiles by children when on mushroom collection trips with their parents [28]. In addition, several mushroom species (e.g., *Ganoderma oerstedii* and *Pycnoporus sanguineus*) have important medical applications. An unidentified bolete mushroom is used for the treatment of heart and joint problems, while *G. oerstedii* and *P. sanguineus* extracts are a medication against skin ailments as well as fever. A drink made from *G. oerstedii* is used as medicine against stomach pain, diarrhea, and kidney complications [28]. In general, the Wixaritari are an example of an indigenous Mexican people who still possess profound knowledge on many aspects of mushroom utilization, which began thousands of years ago in pre-Hispanic times.

## 3. Mexican Microorganisms in Modern-Day Biotechnology

In modern times, several microorganisms (e.g., algae, fungi) have been studied, produced, and processed for an abundance of applications in Mexico. Some of the most relevant species for these purposes are shown in Table 1. 

### 3.1. Algae

The study of algae in Mexico has grown through the years. From 1787 through 1954, there were at least 51 collectors of algae officially recognized through local and international herbaria. Most of the collectors were from North America or Europe [14].

At present, over 5000 species of algae have been identified in Mexico [29,30]. Some algae exported from Mexico since the 1950s are: *Macrocystis pyrifera,* used for alginate extraction; *Gelidium robustum,* used for agar extraction; and *Porphyra perforata,* used for food [31,32]. Baja California is the main area of production for these algae [33].

In the 1990s, there were over 90 marine algae-derived products in Mexico [34]. Algae are used in several different settings. Some algae have antioxidant properties, while others have activity against bacteria, viruses, fungi, nematodes, and pathologies, such as obesity and cancer [35,36,37,38,39,40,41,42,43,44,45,46,47]. In Jalisco alone, there are over thirty species of algae with potential applications in cosmetics, medicine, and food [48]. Algae can be exploited for their bioactive compounds, which may have allelopathic, anti-predator (such as ones produced by diatoms as defenses against freshwater crustaceans who graze on them), and antimicrobial activities [49,50].

Spirulina (*A. platensis*) is one of the more widely used and produced algae. In 1978, there were over 5 tons of spirulina production for algae flour in Mexico [51]. In the 1970s, Texcoco Lake, near Mexico City, began the first large-scale spirulina production operation [52]. In 1982, Texcoco Lake produced over 1 ton of spirulina flour by itself [53]. Texcoco-produced spirulina was shown to have a hepatoprotective effect against fatty livers in rats (as a 5% dietary supplement) [54,55]. Moreover, *A. platensis* was shown to have neuroprotective effects in mice, against 1-methyl-4-phenyl-1,2,3,6-tetrahydropyridine (MPTP) neurotoxicity, and is used as a model in Parkinson’s disease [56].

Diatoms have been grown as in vivo food for aquatic species. Several conditions were studied to increase yield and improve the biochemical composition of the algae. Near Acapulco Guerrero, three species of diatoms were tested, with the greatest cell growth and biomass increase being of *Biddulphia alternans* [57].

At least 24 species of red algae drift periodically to the Yucatan Peninsula. Eighteen marine algae species isolated from Yucatan Peninsula were shown to have antimicrobial activity, with the best results belonging to *Ceramium nitens* [58]. Some of the algae from Yucatan produce carrageenans, and are frequently used in the pharmaceutical, medicinal, and cosmetics industries [59]. *Eucheuma isiforme* showed carrageenan yields between 44.6, and 31.8%, with different extraction methods [60,61].

Other applications of marine algae include the production of vitamin B12. At the coast of Mexico, 31 species were found to produce an average of 0.0827 mg/gr of vitamin B12 [62]. Additionally, the use of a marine algae extract made in Mexico (named ALGAENZIMS) as an agricultural stimulant was capable of increasing the yield of different crops, over one ton per hectare [63].

### 3.2. Fungi

Fungi are relevant for the production of valuable compounds (enzymes, pharmaceuticals, food additives), participate in important ecological processes (biocontrol of pathogenic organisms, waste fermentation), and have ludic uses (e.g., fungi producing hallucinogenic substances). 

#### 3.2.1. Enzyme Production

A Mexican xerophilic strain of *Aspergillus niger* GH1 is a competent producer of the enzyme invertase, i.e., β-fructofuranosidase [64,65]. The invertase gene of strain GH1 was cloned, showing very high homology to invertases from *Aspergillus kawachi* IFO 4308 (93% identity) and *A. niger* B60 (97% identity). Heterologous expression of the invertase-coding gene in the methylotrophic yeast *Pichia pastoris* resulted in the production of a functionally active enzyme, which had an optimum pH and temperature of 5.0 and 60 °C. The specific activity of the protein was 3389 U/mg [65]. This invertase is therefore attractive for the large-scale production of inverted sugar (a mixture of glucose and fructose). In a different study, the authors reported a protocol for optimizing invertase production by the *A. niger* strain GH1, by utilizing cheaper substrates, such as molasses and sugarcane bagasse [66].

There are several more examples in which filamentous fungi isolated from Mexican semi-deserts have been used for the biotechnological production of economically relevant enzymes. One of these is tannase, which is used in the nutritional industry for the production of instant teas, for example [67]. Chemically, tannase hydrolyzes the ester bond that is a characteristic feature of tannins. Several *Aspergilli* were isolated from soils originating from Mexican semi-deserts and characterized regarding their tannase genes by sequencing [68]. The results showed that the catalytic site (GXSXG) is, as expected, highly conserved. *A. niger* GH1 and PSH tannase sequences were found to have an extra codon (glycine). These two sequences are, in evolutionary terms, the oldest ones among the samples studied by Borrego-Terrazas *et al.*, and led to the identification and characterization of genes from strains that are well adapted to extreme environments. This strategy might lead to the production of relevant enzymes that are characterized by increased temperature stability [68].

Xylanase, which has a low-molecular-weight, has been isolated and studied from the Mexican *Aspergillus* sp. strain FP-470 [69]. Biochemical characterization of the enzyme revealed optimum pH and temperature values of 5.5 and 60 °C, respectively. The activity of the xylanase was demonstrated with 4-O-methyl-D-glucuronoxylan as the substrate. The Km (Michaelis constant) of the enzyme was reported to be the Km of 1.9 mg/mL. Cellulose and other polysaccharides were not processed by the xylanase. One possible application of this enzyme is found in bread preparation: the addition of purified xylanase to dough resulted in a substantial increase of bread volume [69].

By using Mexican strains *Aspergillus* sp. FP-180 and *A. awamori* NRRL 3112, it was demonstrated that protoplast regeneration under acidic stress could highly benefit pectinase production [70]. Protoplasts were able to regenerate mycelium at low pH values of 1.7. The resulting acid-adapted *Aspergillus* strains were able to grow at pH values of 1.5 (the original strains were not able to do so). It even showed a two-fold increase in cell growth at pH 2.0 in liquid culture. This capability is very attractive for the production of pectinases because it led to a four-fold production of exo-pectinase and a nine-fold production of endopeptidase [70].

#### 3.2.2. Biocontrol of Pests/Pathogens

*Eichhornia crassipes* (water hyacinth) is considered the most invasive aquatic weed in the world [71]. Biocontrol agents, used to stop massively invasive growth threatening water availability, are therefore in high demand. The fungus, *Cercospora piaropi,* is a well-known pathogen of water hyacinth and is capable of decreasing the growth of its host by promoting a debilitating foliar disease. An important component of *C. piaropi,* to attack water hyacinth, is the red pigment cercosporin, a broad-spectrum phytotoxin [72,73]. A Mexican isolate of *C. piaropi* was evaluated regarding its cercosporin production under light/dark conditions. Interestingly, production of the pigment was also observed without light, but at a reduced level, 72.59 mg/L-continuous light vs. 25.70 mg/L-dark after 31 days of cultivation [71].

In general, fungi can be extremely useful as biocontrol agents. *Metarhizium anisopliae* (family: Hyphomycetes) has been evaluated for controlling the Asian blue tick *Rhipicephalus microplus,* which can be found feeding on cattle in the Mexican tropic forests [74]. Specifically, conidia (asexually generated spores) were being evaluated for their biocontrol usability against the ticks. The entomopathogenic effect begins with the adhesion of the conidia to the cuticle with subsequent germination and the formation of specialized structures, so-called appressoria. These enable the penetration of hyphae through the cuticle and growth inside the animals, causing severe damage to all tick stages [75]. Conidia from two different strains (Ma14 and Ma34) were evaluated [74]. In the adult ticks, the Ma34 strain showed an efficacy of 100% on engorged females at 1 × 10^8^ down to 1 × 10^6^ conidia/mL, which was more effective than Ma14. Egg oviposition was significantly reduced by 55.5% (Ma34) and 39.1% (Ma14 + Ma34 mixture), respectively, compared to control groups. In the larvicidal evaluation, Ma14 showed only a moderate efficacy of 45–62%; however, Ma14 + Ma34 increased it to 90% at a conidia concentration of 1 × 10^8^ conidia/mL. In summary, the fungus *M. anisopliae* is well suited to control *R. microplus* under laboratory as well as field conditions. In a later study, several novel strains were isolated (MaV05, MaV09, and MaV22) that proved to be highly effective (i.e., causing mortality of >90%) for the control of tick populations [76].

Recently, a strategy against the formation of bacterial biofilms by utilizing co-cultivation of the yeast *Rhodotorula mucilaginosa* UANL-001L with *Escherichia coli,* was presented [77]. The yeast produces an exopolysaccharide that can block the growth of individual bacteria and, importantly, is able to inhibit the formation of problematic biofilms in *E. coli*, *Pseudomonas aeruginosa* and *Staphylococcus aureus*. The authors suggest that the mode of action of the exopolysaccharide is based on modification of the bacterial cell wall [77]. In terms of the activity of the exopolysaccharide, concentrations between 1000 and 2500 ppm resulted in up to 60% growth inhibition of *S. aureus*. The compound was also effective against *E. coli* and *P. aeruginosa*, leading to growth inhibitions of 27% and 24%, respectively, at exopolysaccharide concentrations between 2000 and 2500 ppm [77].

Livestock is vulnerable to infection from various parasites, among these, different types of worms. The gastrointestinal nematode *Haemonchus contortus* is especially problematic, leading to enormous economic losses worldwide. Specimens of the edible fungus *P. djamor* strain ECS-0127 collected in southeastern Mexico (Cacaohatán, Chiapas) were recently evaluated for their ability to kill parasitic nematodes [78]. One of the hydroalcoholic fractions (named PdB) collected from the fruiting bodies showed 100% of egg hatching inhibition at 5 mg/mL. Furthermore, larvicidal activity was very efficient, at >97.2% after 24 h at a PdB concentration of 20 mg/mL. Importantly, fraction PdB was also effective at reducing nematode larvae (−92.56%) in artificially infected gerbils (*Meriones unguiculatus*). The authors analyzed fraction PdB by nuclear magnetic resonance and identified allitol and an unidentified terpene. The authors conclude that these compounds could indeed be responsible for the control properties of the extract PdB on the nematode *H. contortus* [78].

#### 3.2.3. Production of Pharmaceuticals

Paclitaxel (brand name Taxol) is a valuable oxygenated diterpene with pharmacological applications [79]. The compound can be isolated from the bark of yew trees of the genus *Taxus*. However, nowadays it can also be produced synthetically [79,80]. A more cost-effective alternative could be production of paclitaxel by fungal fermentation [81]. Solid-state fermentation, but not liquid fermentation, was found to be very effective in the biosynthesis of paclitaxel by the endophytic fungus *Nigrospora* sp. (isolated from the bark of Mexican yew trees), yielding paclitaxel titers of up to 221 ng/L. It is relevant to note that the base medium had to be concentrated eight times to give the most promising results [81].

Mexican fungi have been used for the isolation of compounds that have pharmacological action on the human immune system. One example is the tree parasite *G. oerstedii*, which produces ergosta-7,22-dien-3-one [82]. This compound exerts manifold effects on the immune system, for example, inducing the production of toll-like receptors (which recognize molecular moieties associated with various pathogens), and immune system modulators, such as cytokines, chemokines, nitric oxide, and cellular adhesion molecules. Cytotoxic effects could not be detected on HeLa or J774A.1 (type of macrophage) cells [82]. The compound ergosta-7,22-dien-3-one could therefore have relevant pharmacological applications.

*G. lucidum*, a close relative of *G. oerstedii*, is highly valued for its beneficial effects on human health [83]. In particular, antihyperlipidemic and antioxidant activities of this valuable fungus have been described [84], making it very attractive to combat several metabolic pathologies, such as diabetes and obesity. A recent publication investigated the transcriptome response of liver and kidney tissue from mice (C57BL/6) to a high-cholesterol diet and standardized *G. lucidum* extracts at the transcriptome level [85]. The extracts were isolated from fruiting bodies, which were cultivated on either a conventional substrate or substrate containing acetylsalicylic acid (ASA) as a promoter of fungal growth and secondary metabolite production. Both extracts (especially from ASA-supplemented ones) were capable of modulating the transcriptome, in terms of reducing lipid biosynthesis and enhancing lipid degradation and secretion. Some of the genes that are transcriptionally influenced by the *G. lucidum* extracts correlate with the enrichment of gut bacteria of the genus *Lactobacillus*. This observation is probably based on the fact that the extracts were previously reported to exert prebiotic effects on certain gut bacteria [86]. Overall, Mexican extracts from *G. lucidum* offer interesting perspectives to reduce the risk of hypercholesterolemia-associated metabolic disorders [85].

#### 3.2.4. Food Improvement

*Pleurotus* species are attractive as additives to improve the physicochemical, nutritional, and sensory properties of flours, doughs, and tortillas [87]. *Pleurotus agaves* (traditional maguey mushroom) is highly appreciated as a food supplement in several regions of Mexico. Traditional nixtamalization is a procedure in which corn kernels are boiled in a calcium hydroxide solution, and after soaking for 16 h, the cooked corn, called nixtamal, is ground, thereby forming dough. Ecological nixtamalization is an improved procedure, which reduces water consumption, solid waste production, and environmental pollution. Furthermore, antioxidants, such as anthocyanin and other flavonoid contents, are better preserved. However, tortillas produced with ecological nixtamalization receive low scores in terms of sensory properties, which are important for their acceptance [87]. It was shown that adding 9% ground mushroom to the dough enhances the sensory scores to the same level as for tortillas prepared with traditional nixtamalization [87]. Therefore, authors developed a new environment-friendly product using local traditional foods (tortilla, *P. agaves*) of complementary nutritional value.

Maize (*Zea mays*) ears can be infected by the basidiomycete *Ustilago maydis,* which usually leads to the development of galls, which contain teliospores. These galls are known as “huitlacoche” or “cuitlacoche” in Mexican cuisine and are highly valued by customers [88]. Recently Castañeda de León et al. described the controlled production of “huitlacoche” with the goal to produce edible galls [89]. Tester strains of *U. maydis* with defined mating type loci produced the highest yields of smut galls (>12 t/ha) compared with wild type strains (4.8 t/ha) and hybrids (5.6 t/ha). Still, wild type strains were producing a qualitatively better product of umami and maize, which was less bitter and acidic, but sweeter, of and more to consumer liking. In summary, the authors demonstrate the importance of utilizing the wide genetic diversity of Mexican wild type strains to develop better hybrids yielding improved “huitlacoche” [89].

#### 3.2.5. Waste Fermentation

The filamentous fungus *A. niger* has been successfully used to ferment the Mexican tequila plant *Agave tequilana* waste [90]. During cultivation, the strains CH-A-2010 and CH-A-2016 produce several important enzymes that are secreted into the growth medium. Among these are inulinase and endo-pectinase, which were determined to have activities of 1.48 U/mL and 2.7 U/mL after 120 h of fermentation, respectively. These values are significantly higher than those that were measured for lemon peel, i.e., 0.2 U/mL and 1.75 U/mL [90]. These results demonstrate that the environmentally problematic waste (e.g., burning or dumping leads to air and soil pollution) from *A. tequilana* can form an attractive basis for the fungal production of valuable enzymes with industrial applications.

The tropical white-rot fungus *Trametes maxima* grows on logs or dead trunks in hygrophile or mesophile areas. It recently emerged as a competitive bioremediator of dye-containing wastewaters [91]. The authors studied the decolorization capacity of *T. maxima* LE130 and *Trametes* sp. LA1 in solid and liquid media. Decolorization was especially effective on solid media by the *Trametes* isolates, even superior to the decomposing activity of the white-rot fungus *Phanerochaete chrysosporium* ATCC 24725. The authors identified laccases as the main active factors. Both *T. maxima* LE130 and *Trametes* sp. LA1 produce different isoforms of this enzyme: one protein at 43.9 kDa (*T. maxima* LE130) and three proteins at 52.7 kDa, 58.6 kDa, and 67.3 kDa. Importantly, the liquid phase of *Trametes* sp. LA1 culture was capable of detoxifying different dyes (i.e., anthraquinone dye Remazol Brilliant Blue R, azoic Reactive Black 5, and the triphenylmethane Crystal Violet) without complementation of redox mediators [91]. The properties make fungi of the genus *Trametes* very attractive for the treatment of wastewaters that are contaminated by dyes.

Waste from commercial beverage production is environmentally problematic because it contains many potentially harmful compounds, such as acids, alcohols, nitrogen salts, etc. There is an urgent demand to find ways to lessen the negative impact of these wastes. The production of beverages, such as tequila and Brazilian cachaça, results in the accumulation of waste known as vinasse. Yeasts can tolerate the composition of vinasse better than other microorganisms and are therefore well suited to ferment this type of waste [92]. It was shown that different yeast species (*Candida parapsilosis*, *Pichia anomala*, and *S. cerevisiae*) could aid in the fermentation of vinasse, especially tequila waste, by decreasing the oxygen demand [93]. Furthermore, valuable volatile compounds were formed during fermentation, such as ethyl lactate (non-*Saccharomyces* yeasts) and ethyl hexanoate, which is an important aroma compound (pineapple/green apple). In conclusion, the authors showed that yeast-assisted vinasse fermentation is an economically interesting strategy for the production of value-added products with a concomitant reduction in environmental pollution [93].

**Table 1:** Examples of fungi and algae, and their applications in Mexico.

Species	Field	Use	Reference
FUNGI			
*Amanita basii+*	nutrition	food (supplement)	[28]
*Amanita laurae+*	nutrition	food (supplement)	[28]
*Aspergillus awamori*	biotechnology	pectinase production	[70]
*Aspergillus niger*	biotechnology	invertase production	[64,65,66]
*Aspergillus* sp.	biotechnology	xylanase production	[69]
*Aspergillus* sp.	biotechnology	pectinase production	[70]
*Aspergillus* spp.	biotechnology	tannase production	[68]
*Calvatia cyathiformis*	ludic use	projectile (toy)	[28]
*Candida parapsilosis*	bioprocessing	waste treatment	[93]
*Cercospora piaropi*	environment	pest control (water hyacinth)	[71]
*Ganoderma lucidum*	pharmaceuticals	medical	[85]
*Ganoderma oerstedii*	pharmaceuticals	ergosta-7,22-dien-3-one production	[82]
*Ganoderma oerstedii*	pharmaceuticals	medical	[28]
*Metarhizium anisopliae*	environment	pest control (Asian blue tick)	[74]
*Nigrospora* sp.	pharmaceuticals	paclitaxel production	[81]
*Panaeolus* spp.	ludic use	hallucinogen	[22]
*Pichia anomala*	bioprocessing	waste treatment	[93]
*Pisolithus* spp.	materials	projectile (toy)	[28]
Pleurotus agaves *	nutrition	food (supplement)	[87]
*Pleurotus djamor*	nutrition	food (supplement)	[28]
*Pleurotus djamor*	environment	pest control (nematode)	[78]
Pleurotus opuntiae *	nutrition	food (supplement)	[28]
*Psilocybe* spp.	ludic use	hallucinogen	[22]
*Psilocybe cubensis*	ludic use	hallucinogen	[22]
*Pycnoporus sanguineus*	pharmaceuticals	medical	[28]
*Rhodotorula mucilaginosa*	pharmaceuticals	bacterial biofilm inhibition	[77]
*Saccharomyces cerevisiae*	bioprocessing	waste treatment	[93]
*Stropharia* spp.	ludic use	hallucinogen	[22]
*Trametes maxima+*	bioprocessing	waste treatment	[91]
*Trametes* sp.	bioprocessing	waste treatment	[91]
*Ustilago maydis*	nutrition	food (supplement)	[28]
*Volvariella bombycina*	nutrition	food (supplement)	[28]
ALGAE			
*Arthrospira platensis*	nutrition	food (supplement)	[16]
*Arthrospira platensis*	pharmaceuticals	neuro and hepatoprotective	[56]
*Biddulphia alternans*	pisciculture	food (supplement)	[57]
*Ceramium nitens+*	pharmaceuticals	antimicrobial activity	[58]
*Eucheuma isiforme*	pharmaceuticals	thickener and stabilizer	[60,61]
*Eucheuma isiforme*	nutrition	food (thickener and stabilizer)	[60,61]
*Gelidium robustum*	biotechnology	agar extraction	[31,32,33]
*Macrocystis pyrifera*	biotechnology	alginate extraction	[31,32,33]
*Porphyra perforata*	nutrition	food (supplement)	[31,32,33]

*: species endemic to Mexico; +: species found mostly in North America, Central America, or the Caribbean.

#### 3.2.6. Ludic Uses of Hallucinogenic Fungi

As outlined in Section 2.2, shamanic religious practices involving the use of fungi that produce hallucinogenic substances, such as psilocybin, were common and an essential element of social life in Mexican pre-Hispanic communities. Nowadays, there are discussions in Mexico to classify psilocybin as a type 2 drug (medical purposes) and not as a type 1 drug (no medical use, highly addictive). Several data suggest that psilocybin can be useful in the treatment of mental conditions, such as anxiety and depression [94]. Moreover, psychedelic tourism is a growing phenomenon in Mexico [95]. One of the reasons for this observation is that tourists from main urban centers are increasingly visiting indigenous communities with the desire to take part in religious rituals that involve the use of hallucinogenic substances, e.g., peyote-tourism in Wixárika communities and magic mushroom-tourism in Oaxaca. Furthermore, the increased demand for psychedelic experiences leads to a growth of clinics and retreats that offer their services to tourists [95]. This is problematic because psilocybin is officially illegal to possess, cultivate, and sell in Mexico.

### 3.3. Other Microorganisms

Other eukaryotic microorganisms, which include organisms often grouped as protists, are poorly understood, and there is a lack of awareness about them. It has been established that protists or eukaryotic microbes have been neglected or even forgotten as an important part of ecological studies [96]. When these organisms are ignored at the level of basic science, it can be understood that applied science lags in other areas. One example is what little research has been performed in bioactive molecules regarding protists. No such research was found in organisms specific to Mexico. However, some bioactive compounds have been identified as having enzymatic potential in industrial applications and food supplements, and secondary metabolites with potential in drug discovery. There is mounting evidence about the potential discovery of interesting molecules in protists [97]. One such example comes from social amoebae, where *Dictyostelium discoideum* produces terpene cyclases [98]. It stands to reason that a megadiverse country, such as Mexico, is also rich in protist organisms, with potential bioactive molecules of interest.

## 4. Perspective: Utilizing Mexican Biodiversity for the Improvement of Society

**Figure 1:**
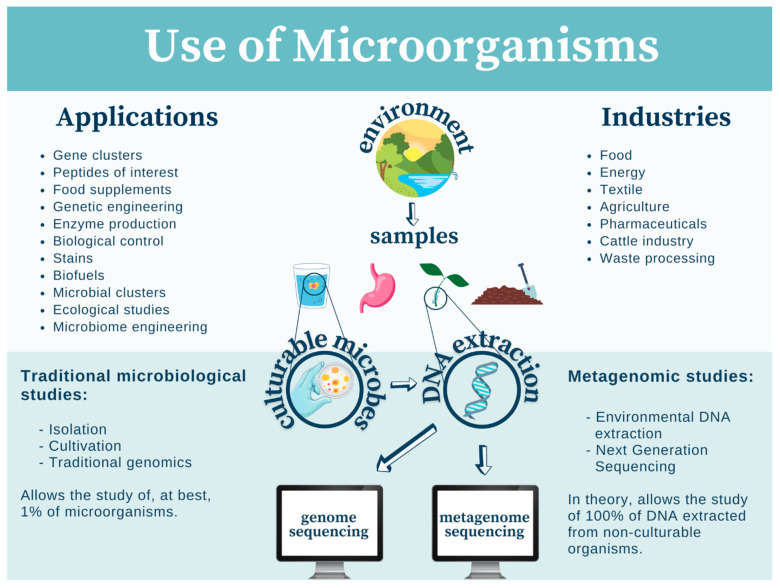
Use and study of microorganisms in modern biotechnology.

As stated previously, Mexican biodiversity offers many relevant opportunities to discover unknown microorganisms and use them for various biotechnological applications (Table 1). As such, the identification, characterization, and application of said microorganisms are expected to improve the field of biotechnology substantially (Figure 1). However, it should not be forgotten that biodiversity hotspots not only offer huge potential, they are also threatened with destruction, due to, for example, environmental pollution and illegal logging, leading to deforestation. 

The list of Mexican biodiversity hotspots includes the San Pedro Mártir and Constitución de 1857 national parks in Baja California, El Pinacate and Alto Golfo de California in Sonora, Reserva de la Biosfera Janos in Chihuahua, Maderas del Carmen in Coahuila and Laguna Madre, the coast cenotes of the Yucatan Peninsula, Reserva de la Biosfera Manantlán in Jalisco, Selva Zoque extending through Veracruz, Chiapas and Oaxaca, and Delta del Río Bravo in Tamaulipas, among others.

Methodologically, traditional microbial studies and metagenomics can be efficiently combined to isolate and study novel and promising microorganisms from environmental samples (Figure 1). Environmental DNA isolation, the construction of genetic libraries, and their subsequent next-generation sequencing can identify new genes and clusters, encoding, for example, non-ribosomal peptide synthet ases and polyketide synthases that often allow the production of unusual and potentially highly interesting compounds, once they are successfully activated under laboratory conditions [99].

This could benefit the research and development strategies in energy, food, and waste processing industries, among several others (Figure 1). Relevant compounds range from new enzymes, pharmaceuticals, food supplements, and many more. Government and private institutions should combine their efforts to make use of the incredible potential offered by the isolation of new microbes from biodiversity hot spots (before it is too late to do so).

The opportunities presented by Mexican microbial diversity, to study and increase our biotechnological knowledge, are vast. Combining and complementing the study of traditional customs with new technologies should allow us to discover new and useful microorganisms, metabolic routes, enzymes, and bioactive compounds that could be attractive in different sectors. This approach is, of course, not limited to Mexico.
